# A biscarbene gold(I)-NHC-complex overcomes cisplatin-resistance in A2780 and W1 ovarian cancer cells highlighting pERK as regulator of apoptosis

**DOI:** 10.1007/s00280-023-04548-1

**Published:** 2023-06-05

**Authors:** Philipp König, Roman Zhulenko, Eloy Suparman, Henrik Hoffmeister, Nico Bückreiß, Ingo Ott, Gerd Bendas

**Affiliations:** 1 Department of Pharmacy, University Bonn, An der Immenburg 4, 53121 Bonn, Germany; 2 Institute of Medicinal and Pharmaceutical Chemistry, Technische Universität Braunschweig, 38106 Brunswick, Germany

**Keywords:** Cisplatin, Chemoresistance, ERK-signaling, Gold(I)-NHC-complex, Ovarian cancer, Thioredoxin reductase

## Abstract

**Purpose:**

Cisplatin resistance is the major obstacle in the clinical treatment of ovarian cancer patients. Molecular mechanisms of cisplatin resistance are multifaceted. Gold(I)-compounds, i.e. *N*-heterocyclic carbene-gold(I)-complexes (NHC-Au(I)) has been regarded as promising cytotoxic drug candidates. However, their potential to overcome cisplatin resistance has hardly been addressed yet. Here we investigated the activity of the gold(I) drug auranofin and the NHC-Au(I)-compound MC3 in W1CR and A2780cis cisplatin-resistant ovarian cancer cells.

**Methods:**

Cytotoxicity of auranofin and MC3 was detected by MTT assay, correlated with intracellular gold(I) content, analyzed by AAS, and with flow cytometric detection of the cell cycle. Insight into cellular redox balance was provided by fluorimetric ROS-formation assay and western blotting thioredoxin (Trx) and Nrf2. The role of ERK was elucidated by using the inhibitor SCH772984 and its impact on cytotoxicity upon co-treatment with cisplatin and Au(I)-compounds, respectively.

**Results:**

MC3 overcomes cisplatin resistance in A2780cis and W1CR, and auranofin in W1CR cells completely, which is neither reflected by intracellular gold levels nor cell cycle changes. Upregulated redox balance appears as a basis for resistance. W1CR cells possess higher Trx levels, whereas A2780cis cells display strong Nrf2 expression as anti-oxidative protection. Nevertheless, overcoming redox balance appears not primary mode of activity comparing cisplatin and gold(I)-compounds. pERK emerges as a critical component and thus a promising target for overcoming resistance, regulating apoptosis differently in response to either gold(I) or cisplatin in A2780 cells.

**Conclusion:**

These data reflect the complexity of cisplatin resistance in cell models and emphasize NHC-Au(I)-complexes as prospective cytotoxic agents for further investigations in that respect.

**Supplementary Information:**

The online version contains supplementary material available at 10.1007/s00280-023-04548-1.

## Introduction

Ovarian cancer possesses the highest mortality rate among gynecological tumors, although it ranks 8th in the incidence of all malignancies in women. The high mortality is related to the fact that it is usually diagnosed in late stages when peritoneal spread or distant metastases have already appeared. The standard treatment includes a maximum cytoreductive surgery followed by a guideline-based cytotoxic treatment using platinum-based drugs or paclitaxel [1]. Cisplatin, despite afflicted with dose-limiting toxic side effects, has an outstanding role in the treatment of ovarian cancer. Although patients respond well in initial treatment, a high recurrence rate remains a major obstacle in the clinical treatment of patients. However, second-line treatment using other drugs, such as topotecan or doxorubicin, cannot solve this problem, since recurrence is often associated with resistance formation. The molecular mechanisms of resistance appear multifaceted and far from being understood.

Cisplatin, established for roughly four decades in the clinics as a DNA-impairing agent, has triggered the search for other cytotoxic metallodrugs. Among them, gold(I) and gold(III) complexes are the focus of research to provide novel cytotoxic agents, differing in targeted mechanisms. Although aurothiolates have a pharmacological history as potential anti-inflammatory drugs, *N*-heterocyclic carbene-gold(I) complexes (NHC-Au(I)) emerged as the most promising cytotoxic agents during the last decade with respect to stability and possibility for derivatization [2]. Meanwhile, a great number of NHC-Au(I)-complexes have been described and investigated in preclinical stages [3]. Those complexes display cytotoxic activity in the low micromolar range against a broad panel of tumor cell lines by different modes of action [4–6]. Although Au(I)-complexes have been shown to interact with DNA, thus comparable to cisplatin [7], their primary mode of action is based on inhibition of the thioredoxin reductase (TrxR) [8, 9]. TrxR is a selenoenzyme belonging to the disulfide oxidoreductase family in mammalian cells. Since TrxR remains a reducing environment by transmitting the electron flux from NADPH to its substrate thioredoxin (Trx), this enzyme possesses a redox control of different signaling pathways [10]. Therefore, interference of Au(I)-compounds with TrxR has strong consequences for upregulating radicals, which can contribute to trigger proapoptotic signaling, e.g. via the ASK-p38-MAPK pathway or via the p38-JNK route [8, 10]. Woolston et al. showed that high levels of redox proteins negatively correlate with progression-free survival in patients with ovarian cancer [11]. Due to their intensified metabolic activity, tumor cells possess a higher redox tolerance and handle higher levels of reactive oxygen species (ROS) [12]. Since cancer cells in particular depend on a stable redox balance, TrxR inhibitors show higher toxicity against cancer cells than against cells from healthy tissues [13].

DNA damages, as induced by cisplatin, have a strong impact on ROS formation in cancer cells [14]. Although several molecular mechanisms of cisplatin resistance in tumor cells appear well elucidated [15], such as deregulation of transporter and intracellular detoxification by glutathione, upregulating the redox status of cells appears to be a dominant mechanism, which is much less understood [16]. Additionally, there are classical stress response pathways like the extracellular signal-regulated kinase (ERK)-MAPK route, which are known to either promote survival thereby mediating chemoresistance [17], or induce apoptosis in some cases [18]. This raises the question of whether Au(I)-complexes are promising means to overcome cisplatin resistance of tumor cells by affecting redox axis or survival pathways. In this context, it is worth mentioning that auranofin, the classical aurothiolate has entered a clinical trial in patients with recurrent ovarian cancer (NCT01747798). Although cytotoxic activities of gold-complexes have been intensively investigated in multiple cancer settings, studies to explicitly overcome resistance are rare [19, 20].

**Scheme 1:**
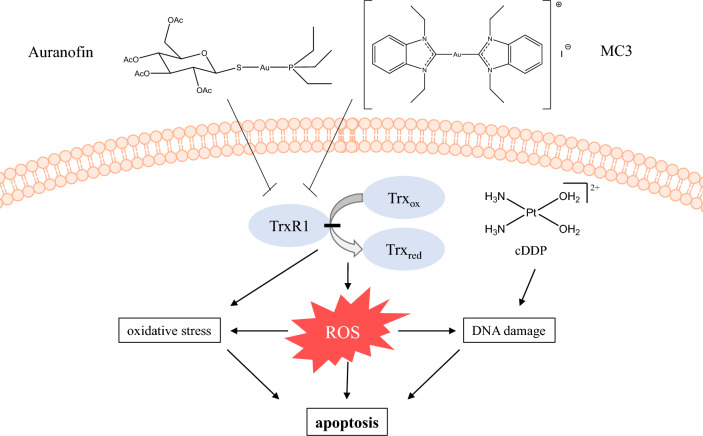
Gold(I) complexes applied and their proposed mode of action. Adapted from [10]

Here we investigate whether auranofin, as an established TrxR inhibitor [21] or the gold(I)complex [di-(1,3-diethylbenzimidazol-2-ylidene)]gold(I) iodide (MC3), recently described [4] have the potency to overcome cisplatin resistance in ovarian cancer cells. The structures are shown in Scheme 1.

MC3 is a cationic organometallic Au(I)-complex with two N-heterocyclic carbene (NHC) ligands, a complex referred to as “biscarbene” in the literature. The compound showed high stability in aqueous and organic solvents, elicited strong cytotoxic, antimitochondrial and immunosuppressive effects, and induced apoptosis through ASK1-p38-MAPK signaling [4, 8, 22]. We used two different ovarian cancer cells lines and their respective cisplatin-resistant subtypes, namely A2780/A2780cis, and the W1/W1CR cell pairs. While the molecular basis of A2780cis resistance has been a matter of multiple investigations, W1CR cells have been much less explored. Recently, it was shown that their cisplatin-resistant state is strongly directed by HSP27 [23].

We can show that both, auranofin and in particular MC3 possess cytotoxicity in the high nanomolar range in all investigated cells, irrespective of cisplatin resistance. Notably, the cytotoxic activity shows no correlation with the intracellular gold levels, suggesting specific interference with resistance targets. Although no obvious differences in affecting the cellular redox balance were evident comparing cisplatin and gold, ERK activation downstream of redox stress processing was elucidated as key and answered the question of why gold-compounds can overcome cisplatin resistance in A2780cis cells.

## Methods

### Gold compounds

[Di-(1,3-diethylbenzimidazol-2-ylidene)]gold(I) iodide (MC3) was prepared and structurally characterized as reported previously [4]. Purity was confirmed by elemental analysis (Flash EA 1112, Thermo Quest CE Instruments) and the measured data for carbon, hydrogen and nitrogen were within 0.4% of the theoretical values. Auranofin was purchased from Sigma-Aldrich GmbH (Steinheim, Germany).

### Cell culture

The human ovarian carcinoma cell lines A2780 and the cisplatin-resistant subtype A2780cis, obtained from ECACC, UK (No. 93112519; No. 93112517-A2780cis); and W1 and cisplatin-resistant W1CR cells (a generous gift by Dr. R. Januchowski, Zielona Gora, Poland) were cultivated at 37 °C and 5% CO_2_ in RPMI 1640 medium containing 10% FCS and 1% penicillin/streptomycin as described before [24]. Cells were detached using a solution of EDTA (0.2 g/L EDTA × 4 Na) for 2 min at 37 °C. All reagents were obtained from PAN Biotech GmbH (Aidenbach, Germany). To ensure the resistance for a longer period, 1000 ng/mL cisplatin (Sigma-Aldrich, Germany) was added to the A2780cis and W1CR cell medium. The maintenance of cisplatin resistance in A2780cis and W1CR cells as well as the absence of mycoplasma in cell culture was confirmed every second week.

### Cytotoxicity assay

The cytotoxicity of cisplatin, auranofin, and MC3 in the indicated cells was determined by MTT assay using 3-(4,5-dimethylthiazol-2-yl)-2,5-diphenyltetrazolium bromide (BioChemica, Applichem GmbH, Darmstadt, Germany) as described [25]. Cells were seeded at a density of 10^4^ cells/well (W1 and W1CR) respective 2 × 10^4^ cells/well (A2780 and A2780cis) in triplicates in 96-well plates (Sarstedt AG & Co, Nümbrecht, Germany). The next day, cells were supplemented with either a dilution series of cisplatin (10^−3.5^ to 10^−7.5^ M) or auranofin (10^−4.5^ to 10^−8^ M) or MC3 (10^−4.5^ to 10^−8^ M). 72 h after the addition of cytostatics, MTT solution (20 µL, 5 mg/mL) was added for 1 h at 37 °C and 5% CO_2_ until formazan crystals were formed. The supernatant was removed and cells were dissolved in 200 µL DMSO. Absorption was analysed at 570 nm, with background subtraction at 690 nm, using a plate reader (Thermomultiscan EX, Thermo, Schwerte, Germany). To examine the effect of sub-toxic gold-compounds on cisplatin resistance, cells were pretreated for 4 h with auranofin at 0.1 µM or MC3 at 10 nM or DPBS (PAN Biotech GmbH) followed by a dilution series of cisplatin. For the calculation of combination index (CI) the software CompuSyn^©^ (ComboSyn, Inc., Paramus, NJ, USA) and the Chou–Talalay method were used to identify whether gold-compounds either act synergistic (CI < 1), additive (CI = 1) or antagonistic (CI > 1) with cisplatin [26]. For combined inhibitor experiments, cells were pretreated for 4 h with either a solution of ATM inhibitor AZD1390 at a concentration of 2.5 µM, or ATR inhibitor elimusertib at 10 nM, or CHK1 inhibitor SCH900776 at 1 µM, or WEE1 inhibitor adavosertib at 10 nM, all purchased from Selleck Chemicals (Houston, TX, USA), or ERK 1/2 inhibitor SCH772984 at 0.1 or 1 µM (Hycultec, Beutelsbach, Germany) followed by a dilution series of auranofin or MC3.

### Cellular uptake of gold compounds

Cells were seeded out in cell culture flasks and incubated with auranofin (0.1 µM) or MC3 (10 nM). After 72 h, the medium was discarded and cells were washed once in cold DPBS. After removing DPBS, cells were detached with EDTA for 2 min and resuspended in fresh medium and centrifuged at 1580×*g* and 4 °C for 4 min. The cell pellet was resuspended in 1 mL DPBS. The remaining suspension was centrifuged again, and the supernatant was removed. This washing step was repeated a second time. Finally, the cell pellets were stored at − 20 °C until further processing. The gold content of the cell pellets was determined using a high-resolution continuum source atomic absorption spectrometer (HRCS-AAS, ContrAA700, AnalytikJena AG, Jena, Germany) according to a method described in detail recently [27].

### Intracellular ROS formation assay

The fluorimetric ROS assay MAK143 (Sigma Aldrich) was performed to examine the intracellular amount of ROS in the indicated cell lines. 1.5 × 10^4^ cells/well were seeded out in black Cellstar^®^ 96-well plates (Greiner Bio-One GmbH, Frickenhausen, Germany) and treated with cisplatin, auranofin or MC3 in either subtoxic concentrations (1 µM of cisplatin, 0.1 µM of auranofin and 0.01 µM of MC3) or at IC_50_ of the corresponding cell line using DPBS as control. The assay was performed according to the manufacturer’s instructions. Fluorescence was analysed using a FLUOstar™ OPTIMA fluorescence scanner (BMG Labtech GmbH, Offenburg, Germany), with a 485 nm excitation and 520 nm emission after 4 h and 24 h of incubation. Finally, the ratio of total ROS measurement after 24 h and 4 h was calculated for each cell line and treatment.

### Western Blot

Cell protein lysate was obtained using cell extraction buffer (Life Technologies, Carlsbad, CA, USA) followed by incubation for 30 min, at 4 °C, on a shaker. After centrifugation, the supernatant was submitted to protein quantification by a BCA Protein Assay Kit. SDS-Page and Western blots were performed as described using stain-free gels [28]. Membranes were incubated with mouse anti-GAPDH (GeneTex, Irvine, USA), rabbit anti-pERK 1/2, rabbit anti-Trx1 (Cell Signaling Technology, Frankfurt am Main, Germany), rabbit anti-ERK 1/2, rabbit anti-Nrf2, mouse anti-α-Tubulin (Santa Cruz Biotechnology, Heidelberg, Germany) as well as goat anti-rabbit and anti-mouse IgG kappa binding protein IgG HRP-conjugated (Santa Cruz Biotechnology) diluted in 1% BSA solution. Western blots were quantified via chemiluminescence using a Clarity Western ECL substrate chemiluminescence kit (BioRad Laboratories GmbH, Munich, Germany). Besides the loading controls GAPDH and α-tubulin, we also used stainfree total protein normalization. Membranes were photographed and analyzed using a ChemiDoc XRS + imaging acquiring system (BioRad) and Image Lab software v. 6.0 (BioRad).

### Cell cycle analysis

Cells were seeded out in cell culture flasks and incubated overnight. The next day, cells were treated with either cisplatin at a concentration of 1 and 2 µM, or auranofin at 0.1 and 1 µM or MC3 at 0.01 and 0.1 µM or DPBS for 24 h. Then, 10^6^ cells were collected and washed once with DPBS, fixed adding ice-cold 70% ethanol dropwise to the cells and incubated at 4 °C for 30 min. Samples were rehydrated with DPBS and incubated with 50 µg/mL RNAse (Thermo Fisher Scientific Inc., Waltham, USA) at room temperature for 15 min followed by incubation with PI (Thermo Fisher Scientific Inc) at a concentration of 3 µM for 15 min in the dark. Cells were analysed by Guava^®^ easyCyte HT 11 Flow Cytometer (Luminex Corporation, Austin, TX, the USA) and FlowJo™ v10.5.3 Software (BD Life Sciences, Franklin Lakes, NJ, the USA).

### Statistical analysis

Cytotoxicity was determined by nonlinear regression and a four-parameter logistic equation with variable hill slope. Inflexion point of the sigmoidal curve was used to calculate IC_50_ values. Comparisons were performed using Prism™ (GraphPad Software, San Diego, CA, USA). Statistical analysis was performed by unpaired t-test or one-way ANOVA following Tukey’s test for multiple comparisons. To check for significance in cellular gold uptake, an unpaired t-test with Welch’s correction was used. For statistics of cell cycle- and ROS assay each sample was compared with the untreated population using one-way ANOVA following Dunnett’s test. To test the significance of Western blots of the cellular comparison of Nrf2 and Trx expression, an unpaired t-test was performed. For statistical analysis of Western blots one-way ANOVA following Tukey’s test for multiple comparisons was used (asterisks indicate *p < 0.05; **p < 0.01; ***p < 0.001; ****p < 0.0001).

## Results

### Auranofin and MC3 can overcome cisplatin resistance acting independently from cisplatin

**Fig. 1:**
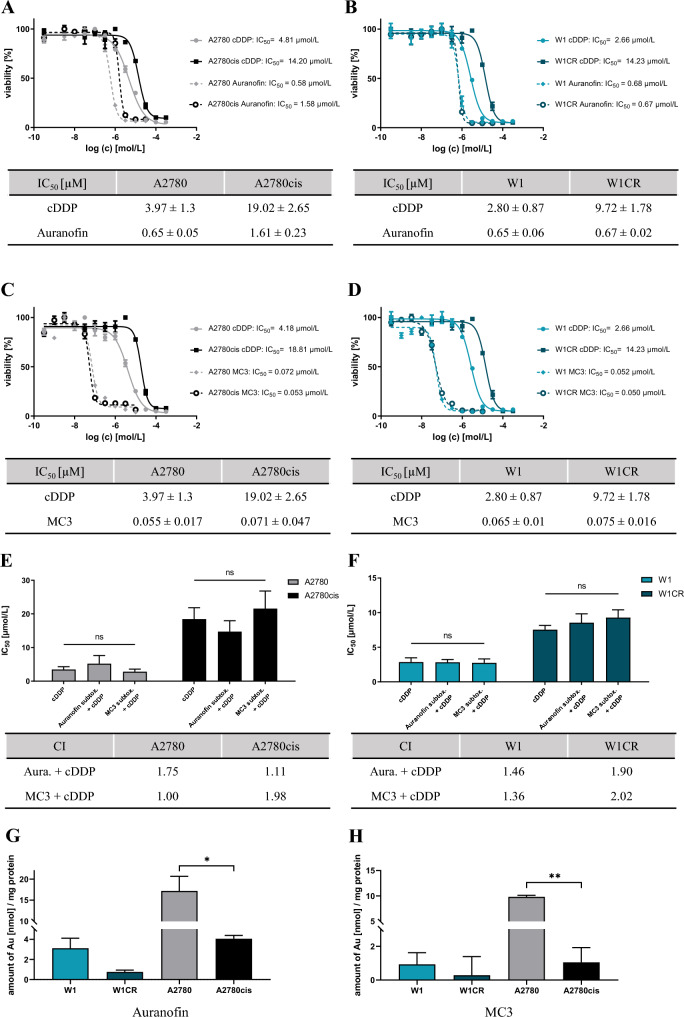
Exemplary cytotoxicity-curves of auranofin in A2780/cis cells (**A**) and in W1/W1CR cells (**B),** and of MC3 in A2780/cis cells (**C**) and in W1/W1CR cells (**D**) compared to cisplatin display an overcoming of cisplatin resistance; tables below curves show means ± SD of biological and technical triplicates. A cellular pretreatment with gold compounds at sub-toxic concentrations prior to cisplatin treatment indicates no impact on cisplatin activity and refers to a different mode of action of cisplatin and gold compounds in at least *n* = 3 biological and technical samples ± SD. Tables below histograms show the resulting CI at the IC_50_ of the combinational treatments (**E** and **F**). Intracellular gold levels of W1/CR and A2780/cis cells after treatment with 0.1 µM auranofin (**G**) and 0.01 µM MC3 (**H**) for 72 h analyzed by AAS indicate no correlation of intracellular gold concentrations and cytotoxicity for overcoming resistance. Shown data represent *n* = 3 samples ± SD, asterisks indicate statistical significance *p < 0.05; **p < 0.01

The cisplatin resistance of A2780cis ovarian cancer cells is indicated by a roughly four-fold higher IC_50_ value of cisplatin compared to its cytotoxicity in A2780 cells (Fig. 1A, C). However, auranofin displays a higher cytotoxicity than cisplatin and partly overcomes the resistance in A2780cis cells, indicated by IC_50_ values of 1.61 vs. 0.65 µM comparing A2780cis vs. A2780 cells (Fig. 1A). Notably, MC3 possesses a more than tenfold higher activity than auranofin, which is identical in both cell lines (Fig. 1C) giving no sign of resistance against this drug in A2780cis cells. A similar picture is evident in W1/W1CR cells, in which auranofin totally overcomes W1CR resistance, displaying cytotoxicity in the high nanomolar range (Fig. 1B), while MC3 again possesses tenfold higher activities (Fig. 1D).

To get a first insight into the probable mode of action of how Au(I)-compounds overcome cisplatin resistance in A2780cis and W1CR cells, a combinatory treatment was performed. All cells were pretreated with Au(I)-compounds at sub-toxic concentrations before cisplatin toxicity was determined. Neither in the A2780 (Fig. 1E) nor in the W1 cell pair (Fig. 1F), pretreatment with Au(I)-compounds affects cisplatin activity. For a more validated insight, combinatory indices (CI) were determined according to the method of Chou and Talalay [26]. The CI of 1 or close to 1 argues for an additive effect of both compounds in these cases. Notably, the resulting CI at the level of IC_50_ in combination with gold and cisplatin indicates that they act either antagonistic. The only exceptions are MC3-pretreated A2780 cells and auranofin-pretreated A2780cis cells, where a slight decrease of IC_50_ compared to the cisplatin control was detected. These data refer that Au(I)-containing drugs and cisplatin neither share a similar mode of action nor act synergistically.

To further elucidate whether the high activity of auranofin and MC3 in all cells is a matter of high intracellular gold levels, thus circumventing probable drug uptake or efflux resistance phenomena, we detected intracellular gold levels by AAS. Notably, both auranofin (Fig. 1G) and MC3 (Fig. 1H) were contained by only 25% or even less in the resistant cells when compared to the sensitive subtypes. This clearly indicates that overcoming resistance in A2780cis and W1CR cells is not related to higher intracellular gold levels, or at least equal ones in the indicated cell pairs. However, the fact that gold(I) is much less contained in the resistant cells refers to uptake restrictions or to efflux transporters. Nevertheless, since these phenomena are not directly related to activity restrictions here, it needs to be further investigated elsewhere.

Taken together, since auranofin as well as MC3 are more cytotoxic than cisplatin and overcome cisplatin resistance in both cell lines not related to intracellular drug levels, further insight into the activity of gold-compounds has to be provided to understand these phenomena.

### Upregulated redox-status is an issue of cisplatin resistance in A2780 and W1 cells and targeted by both, gold-compounds and cisplatin

Although their detailed mechanistic pathways are quite different, cisplatin and gold compounds have in common to induce oxidative radicals intracellularly as a dominant part of their cytotoxicity. Thus, it appears likely that resistance formation is associated with a higher tolerance against oxidative stress.

To tackle this issue, we first determined the ROS formation in a treatment-relevant time frame in the cell lines, expressed as a 24 h/4 h ratio. Cells were compared when untreated or upon treatment with auranofin, MC3, or cisplatin, each at sub-toxic concentrations, or upon toxic stress at the individual IC_50_ values.

**Fig. 2:**
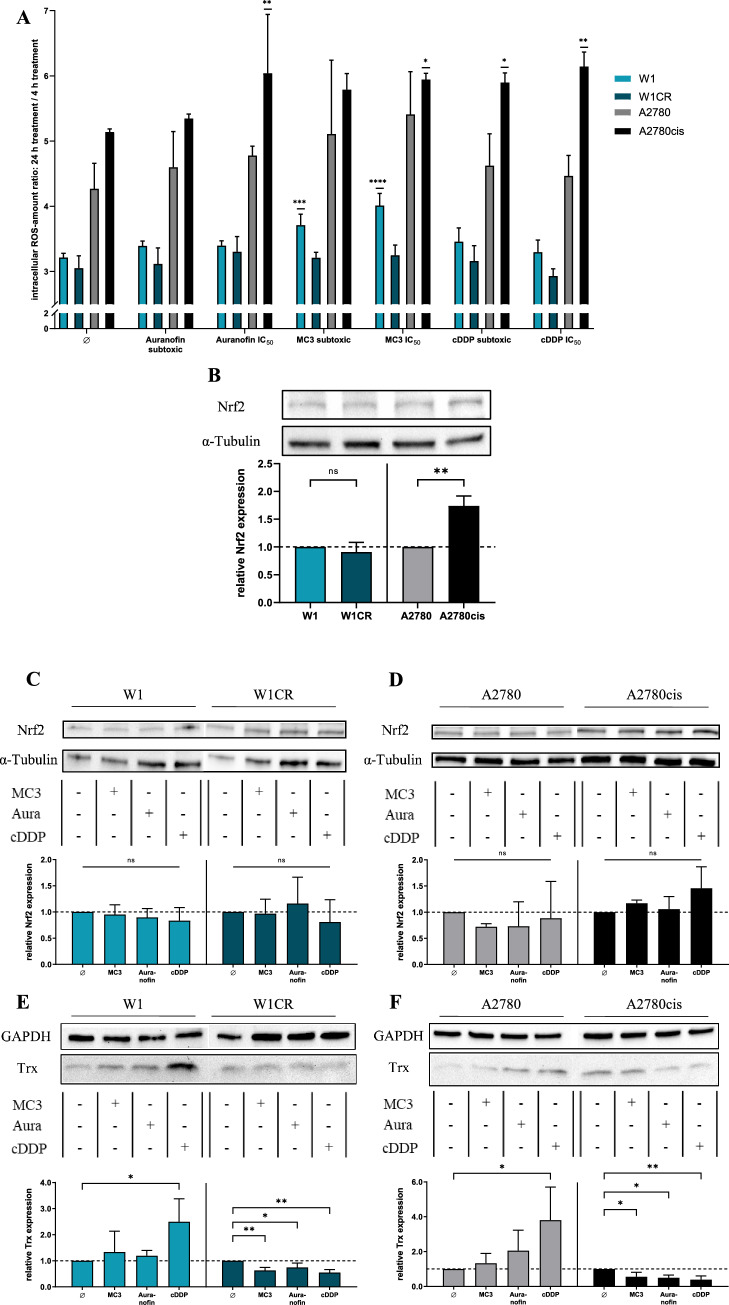
Intracellular increase of ROS as a quotient of 24 h and 4 h measurement as *n* = 4 ± SD (**A**). Comparison of the relative Nrf2 expression in untreated cell pairs normalized to W1 or A2780 cells, respectively (**B**). Relative Nrf2 expression in W1/W1CR cells (**C**) or A2780/A2780cis cells (**D**) upon treatment normalized on untreated cells, respectively. Relative Trx expression in W1 and W1CR cells normalized on untreated cells (**E**), and of A2780 and A2780cis cells normalized on untreated cells (**F**) using 1 µM cisplatin, 0.1 µM auranofin and 0.01 µM MC3. All Western Blot data represent means ± SD of at least *n* = 3 biological samples. Asterisks indicate statistical significance *p < 0.05; **p < 0.01; ***p < 0.001; ****p < 0.0001

It is clearly indicated that untreated A2780 cells have higher upregulation of ROS (roughly five-fold) compared to W1 cells (appr. three-fold) in the identical time range (Fig. 2A). Notably, the cisplatin-resistant A2780cis cells display an even stronger intrinsic upregulation of ROS than A2780 cells, while W1CR and W1 cells do not differ in their basal ROS levels. Upon the different treatments, A2780cis cells display the most remarkable increases in ROS, which reaches significance except the sub-toxic doses of the two gold compounds. Furthermore, MC3 appears to be most active in inducing ROS, in particular inducing a highly significant increase in W1 cells and a significant rise in A2780cis cells at IC_50_ concentration. In contrast, the cisplatin-resistant W1CR and the sensitive A2780 cells do not respond to a significant ROS formation. These data give rise to the assumption that handling oxidative stress is a common issue for the different tumor cells. However, the ROS data do neither allow to derive differences in the mode of action of gold(I)-compounds and cisplatin, nor further insights into the underlying mechanisms of resistance in the two cell pairs.

To follow how the individual behavior of the different cell lines in ROS formation is reflected in the downstream regulation of oxidative stress, in especially addressing differences between the two cisplatin-resistant cell lines, we checked the expression levels of nuclear factor erythroid 2-related factor 2 (Nrf2). Nrf2 is known as a key regulator in cellular processing and protection against oxidative stress [29]. To illustrate the capacity of the cisplatin-resistant cell lines to express Nrf2, the data related to the individual housekeeping protein α-tubulin were directly compared and referred to the intensity of W1 or A2780 cells, respectively, set as the relative value of 1 (Fig. 2B). Data indicate significantly higher levels in A2780cis than in A2780 cells, while cisplatin resistance in W1CR cells is not reflected by increased Nrf2, which shows comparable levels as in W1 cells. We further focused on the Nrf2 expression levels upon cytotoxic treatment in both cell pairs. While Nrf2 is barely affected by the different treatments in both, W1 and W1CR cells (Fig. 2C), the expression of Nrf2 is slightly but not significantly increased in cisplatin-treated A2780cis, but not in A2780 cells (Fig. 2D) upon treatment. This explains that the high oxidative stress in A2780cis cells, indicated by the ROS formation upon cytotoxic treatment in Fig. 2A is antagonized by upregulating the antioxidative functions of Nrf2.

To have a closer look at redox balance and probable differences upon the two cell pairs, we searched upstream in the cellular redox control processing (Scheme 1) and focused on the role of TrxR, which has been described as the primary target of Au(I)-compounds [8]. Blocking TrxR diminishes the turnover of oxidized Trx and thus disturbs the redox balance. Notably, while resistant W1CR display significantly higher Trx levels than the sensitive W1 counterparts, the difference between A2780cis and A2780 cells is only marginal (Supplement Fig. 1). This indicates that redox balance in W1CR cells is dominantly processed via the Trx system in contrast to the Nrf2-driven process in A2780 cells. However, when treated with cytotoxic compounds, differences between the sensitive and the resistant cell lines became evident. While the compounds, i.e., cisplatin induces a significant upregulation of Trx in W1 and A2780 cells (Fig. 2 E,F), regarded as a cellular counter regulation, in particular both resistant cells seemingly display an exhausting of Trx upon treatment.

These data clearly confirm that the cell lines make use of a higher tolerance against oxidative stress as part of their resistance phenomena, although differences exist between W1 and A2780 cells. Notably, the Trx system appears intrinsically more active in the W1CR cells than in the A2780 cell system. It thus can be considered as the target for the gold(I) compounds in cytotoxicity, which can also circumvent the mechanisms of cisplatin resistance in W1CR cells. In contrast, upregulated Nrf2 stands for the higher redox balance activity in A2780cis cells, which can probably compensate for the exhaustion of Trx. However, the findings cannot decipher differences in the activity of Au(I)-compounds and cisplatin in the A2780 cell system to break their increased redox balance and thus, overcome resistance. Additionally, the poorer response of A2780cis cells to treatment with auranofin cannot solely be related to the redox status and needs to be further investigated.

### pERK emerges as key regulator of apoptosis in response to cisplatin and Au(I)-compounds

**Fig. 3:**
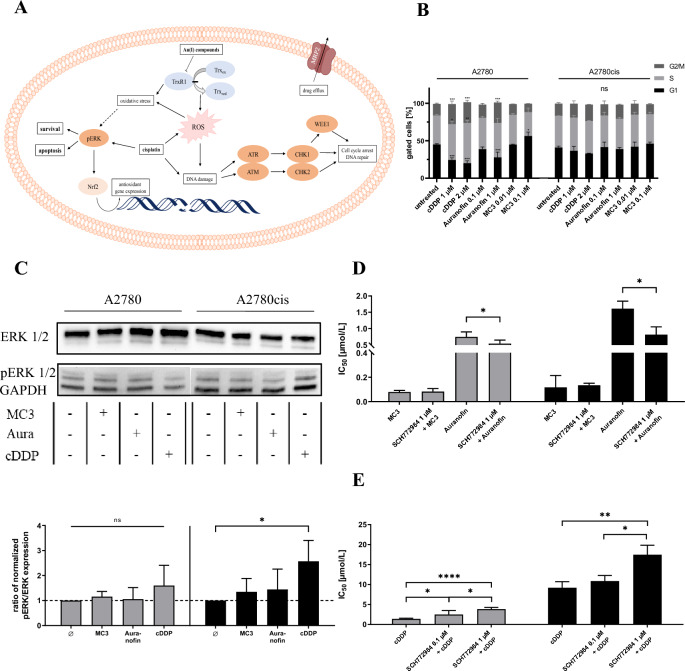
**A** Overview of investigated signaling pathways. **B** Flow cytometric analysis of cell cycle in A2780 and A2780cis cells upon the indicated treatments with cisplatin (cDDP), auranofin, or MC3. **C** The expression of ERK and pERK in untreated A2780 and A2780cis cells and the effects of cytotoxic treatment using 1 µM cisplatin, 0.1 µM auranofin and 0.01 µM MC3, indicated by a representative blot (above) and as calculated ratio pERK/ERK normalized to untreated A2780 or A2780cis cells, respectively (below). Statistical analysis was performed by one-way ANOVA following Dunnettt’s test. **D** The impact of blocking ERK by sub-toxic concentrations of SCH772984 on the cytotoxicity of Au(I)-compounds in A2780 cells (grey) and A2780cis cells (black); and dose-dependently on the cytotoxicity of cisplatin (**E**). All shown data represent means ± SD of at least *n* = 3 biological samples, asterisks indicate statistical significance *p < 0.05; **p < 0.01; ***p < 0.001; ****p < 0.0001

To further elucidate how cisplatin and Au(I)-compounds differ in their activity in A2780cis cells, we checked most downstream (Fig. 3A) whether differences in the cell cycle are evident among the compounds and compared to A2780 cells. Flow cytometric analysis of the cell cycle indicates no differences in untreated A2780 and A2780cis cells (Fig. 3B). However, when treated with the indicated concentrations of the drugs, A2780 cells respond to cisplatin and higher auranofin concentration by a significant shortening of the G1 phase in favor of the G2/M phase. Notably, treatment of A2780cis cells induces no remarkable change in the cell cycle and thus, no differences between the efficiency of cisplatin and Au(I)-compounds.

DNA repair capacity is an issue of antagonizing cytotoxic activities. The enzymatic cascade induced by handling mono- and double-strand breaks has been regarded as a promising target for supporting the cytotoxic activities of cisplatin in ovarian cancer cells [30–32]. Since NHC-Au(I) complexes can interact with DNA [6, 33], we proposed an activation of the DNA damage response pathway in the cells. To figure out whether gold(I) acts at this level upstream of the cell cycle control, and whether blockade of repairing enzymes ATM and ATR, or checkpoint kinases (CHK) affect gold(I) activity (Fig. 3A), we detected whether the IC_50_ values of auranofin and MC3 in A2780 and A2780cis cells were affected, expectedly decreased upon pretreatment with blockers of ATR (elimusertib), ATM (AZD1390), Wee1 (adavosertib), or CHK1 (SCH900776), each at non-toxic concentrations (Supplement Fig. 2). Remarkably, the co-treatment of cells with the indicated inhibitors has no impact on the cytotoxicity of either auranofin or MC3, indicating that these enzymes are neither targeted nor that they restrict gold(I) activities.

Since the pathway of DNA damage repair is obviously not making the difference to cisplatin, we checked ERK activation as direct survival pathway antagonizing oxidative stress. Furthermore, the ERK-MAPK signaling pathway has been associated with an increased translocation of Nrf2 to nucleus mediating the expression of antioxidant proteins [29], making ERK an interesting target in A2780 cells.

Under cytotoxic stress, the total ERK-expression remains relatively unaffected in both cells. Notably, treatment with cisplatin significantly upregulates pERK more than twofold in A2780cis cells, while the gold-compounds induced no increase in A2780 cells and a lower increase than cisplatin in A2780cis cells (Supplement Fig. 3). Calculated ratio of pERK and ERK expression shows a similar trend (Fig. 3C). This suggests that upregulating ERK activity is primarily an intrinsic response of A2780cis cells to antagonize cisplatin cytotoxicity, while gold(I)-compounds either induce a lower activation or suppress this response more upstream, probably at the TrxR activity.

To further follow this postulation, we checked the impact of blocking ERK on the cytotoxic behavior of both, cisplatin and Au(I)-compounds. Therefore, we pretreated the cells with the ERK inhibitor SCH772984 at sub-toxic concentration of 0.1 µM or 1 µM before analyzing the IC_50_ values of the drugs. MC3 cytotoxicity is not affected by additional blocking ERK. The IC_50_ value of auranofin is slightly decreased when co-treated in A2780 cells, and stronger decreased in A2780cis cells (Fig. 3D). These findings give rise to speculate that the slight resistance to auranofin in A2780cis cells, shown in Fig. 1A, is a matter of remaining ERK activity upon auranofin. In contrast, the toxicity of cisplatin is significantly decreased indicated by an increase of IC_50_ values in both cell lines in a concentration-dependent manner when pretreated with SCH772984 (Fig. 3E). This refers to a rather pro-apoptotic role of ERK in cisplatin-treated cells. These findings highlight ERK activity as an indicator to elucidate differences between Au(I) and platinum activity.

## Discussion

Gold compounds attracted much attention as promising drugs, able to affect intracellular signaling pathways with multiple pharmacological consequences. However, their prospects as cytotoxic agents in treating cancer cells are related to blocking of TrxR which affects the cellular redox balance [10]. Although gold compounds represent a new generation of metallodrugs, direct comparative studies of gold compounds with cisplatin, the most prominent metallo-complex in oncology, are rare. The potential of Au(I)-containing drugs to overcome cisplatin resistance has only been investigated in a few studies, e.g. by Messori et al. who also investigated NHC-gold(I) complexes in cisplatin-resistant A2780 cells [34]. Additionally, the antiproliferative effects of auranofin and some analogues was studied in these cells [35]. We aimed to directly compare two different generations of gold compounds: the highly active NHC-gold(I) complex MC3 and the traditional aurothiolate auranofin, which has already shown anti-tumor activity in previous studies.

Our findings indicate that both Au(I)-compounds are highly effective in A2780 and W1 ovarian cancer cells and even in their cisplatin-resistant subtypes, overcoming chemoresistance with similar IC_50_ values. Interestingly, A2780cis cells are more tolerant to treatment with auranofin than the other three cell lines, yet auranofin was still ten times more effective than cisplatin in these cells. MC3 shows the highest potential to affect tumor cells, shifting the IC_50_ values to a low nanomolar range.

Surprisingly, these effects seem to be independent of intracellular gold amounts since the resistant subtypes display much lower gold levels, while the toxicity is almost the same as in wild-type cells. Januchowski et al. reported that cisplatin-resistant A2780 cells upregulate mRNA encoding the ABC-transporter MRP2, which is known to export a broad spectrum of drugs out of cells thereby inducing drug resistance [36]. Apparently, MC3 is recognized by MRP2, or unknown other efflux transporters, efficiently reducing intracellular MC3 levels. However, this does not affect the high activity of this drug, which has similar toxicity in A2780 and A2780cis cells. We have recently shown that W1CR cells do not overexpress MRP2. Additionally, W1CR cells display strongly increased levels of glutathione, which contribute to detoxify cytostatic drugs and thus could explain the lower amount of gold in these cells [28]. Further investigations are needed to understand the interplay of transporters and NHC-Au(I) complexes, but this does not seem to be directly essential here for understanding the activity in overcoming resistance.

Although the indicated cytotoxicity data suggest similarities in the resistance mechanisms of A2780cis and W1CR cells, further insight into the cellular response to gold and cisplatin highlights strong differences in the underlying molecular processes. A ROS formation assay revealed that A2780/cis cells have intrinsically higher oxidative stress than the W1/CR cells. While resistant A2780cis cells respond to cytotoxic treatments with further, significant ROS upregulation, resistant W1CR cells show no deregulation upon treatments and remain at much lower ROS levels. This highlights differences in the molecular pathways of cisplatin resistance in both cell pairs. Although MC3 was most effective in inducing ROS, i.e. significantly in W1 cells, these data do not allow to decipher differences in the cytotoxic activity mode of cisplatin and gold(I)-compounds. However, increasing ROS levels upon treatment with auranofin appear in line with recent data. It has been described that auranofin induces an upregulation of glutathione metabolism thereby increasing intracellular levels of its reduced form (GSH) in A2780 cells. Additionally, an increased uptake of cysteine from the cell medium has been detected. These observations provide an explanation for the slight increase of ROS upon inhibition of TrxR [37, 38]. At least, a certain correlation between ROS induction and cytotoxic activity is evident when considering MC3 with its highest potential to induce oxidative stress, even at the lowest concentration.

Looking more closely at the intracellular redox balance, a more dominant Trx expression and activity appears relevant for W1CR cells, while A2780cis cells use Nrf2 as a key regulator of the anti-oxidant response. Consequently, blocking TrxR by the gold(I)-compounds appear the primary mode of action in W1 and W1CR cells and can therefore be considered as dominant activity in overcoming cisplatin resistance in these cells. In contrast, Trx balance is less emphasized and active in A2780cis cells, so disrupting Trx turnover by gold(I)-compounds cannot solely explain overcoming cisplatin resistance in A2780cis cells. Therefore, Nrf2 as a key regulator of anti-oxidant response appears more informative in these cells. For a better understanding of the stress response in A2780 cells, an Nrf2-knockdown should be considered in future studies.

The impact of oxidative stress and damaged DNA on the cell cycle is more evident in A2780 cells, which is shown by an arrest at G2/M phase when treated with auranofin or cisplatin, and prolonged G1 phase at high concentrations of MC3, resulting in reduced proliferation. In contrast, A2780cis cells are not affected in their cell cycle regulation when exposed to the compounds. However, these data underline different modes of action between the two gold compounds. The G2/M arrest induced by auranofin is similar to the effect of cisplatin and contributes to an explanation for the higher tolerance of A2780cis cells to the aurothiolate, while the sensitivity to the NHC-gold(I) complex is comparable to the other cell lines.

Inhibition of the DNA damage response pathway did not lead to a further sensitization against Au(I)-compounds concluding that increased ROS formation and oxidative conditions do not impair genetic stability. This stands in contrast to cisplatin that forms DNA-adducts thereby inducing repair mechanisms and in consequence acts synergistically with these types of inhibitors [32].

To finally elucidate how gold(I) overcomes cisplatin resistance in A2780cis cells, we focused on the classical MAPK-survival pathway upstream of increased Nrf2 activity, which is known to either induce or to prevent apoptosis in a broad spectrum of cancer cells [39, 40]. Significant upregulation of pERK expression in A2780cis cells upon treatment with cisplatin, but not with the gold(I)-compounds correlates with the observed Nrf2 levels under these conditions. The outcome of ERK-inhibition prior to treatment with cisplatin or gold highlights the different modes of action of these compounds and the key role of ERK/MAPK-signaling in the prevention or promotion of apoptosis.

In detail, MC3 possesses an impressive cytotoxic activity in all investigated cell lines, which is not further enhanced by a simultaneous pERK inhibition. This indicates that pERK is less relevant due to the upstream effects of MC3. Furthermore, the key role of pERK in resistance becomes evident by the auranofin data indicating that remaining ERK activity explains partial resistance in A2780cis cells. Increased toxicity of auranofin when pretreated with SCH772984 offers slight differences between auranofin and MC3. These differences closely follow the cytotoxicity of both compounds in A2780cis cells, i.e. attenuating pERK emerges as key for overcoming resistance phenomena. In general, data indicate that ERK activity plays an anti-apoptotic role in response to stress induced by TrxR inhibition especially in auranofin-treated A2780cis cells.

On the other hand, cytoprotection through ERK-inhibition in combination with cisplatin points out its opposite function. One might conclude that DNA damage induced by cisplatin, but not by gold(I) triggers prolonged ERK-activation and thus a pro-apoptotic activity [41]. This turns out pERK as the apoptosis key regulator in the A2780cis cells, and the dominant difference in response to either gold or cisplatin. This shift in activity could be due to the way the compounds affect the redox balance upstream, and additionally from the intensity of pERK expression. In contrast, considering the ROS formation generated by all of the three investigated compounds solely cannot explain the role in resistance alone.

In summary, these findings confirm that there is no generalizable mode of action when comparing auranofin and NHC-gold(I) complex MC3. However, they act highly effective in cisplatin-resistant ovarian cancer cells. Further investigations are needed to transfer the presented data into practice through in vivo studies with special regard to side effects, to finally consider gold treatment for recurrent tumor patients who no longer respond to standard therapy.

## Supplementary Information

Below is the link to the electronic supplementary material.

Supplementary file1 (DOCX 199 KB)

## Data Availability

The datasets supporting the conclusions of the indicated findings are inserted in the figures or can be requested in data details from the authors.
