# Supplementary material for: A biscarbene gold(I)-NHC-complex overcomes cisplatin-resistance in A2780 and W1 ovarian cancer cells highlighting pERK as regulator of apoptosis
Source: Cancer Chemother Pharmacol. 2023 Jun 5;92(1):57–69. doi: 10.1007/s00280-023-04548-1 (PMC10261188; doi:10.1007/s00280-023-04548-1)
Supplement: Supplementary file 1 — Supplementary file1 (DOCX 199 KB) [file 280_2023_4548_MOESM1_ESM.docx]

**Supplementary Material:**

**A biscarbene gold(I)-NHC-complex overcomes cisplatin-resistance in A2780 and W1 ovarian cancer cells highlighting pERK as regulator of apoptosis**

**Philipp König^1^, Roman Zhulenko^1^, Eloy Suparman^1^, Henrik Hoffmeister^2^, Nico Bückreiß^1^, Ingo Ott^2^, Gerd Bendas^1^**

*^1^Department of Pharmacy, University Bonn, 53121 Bonn, Germany.*

*^2^ Institute of Medicinal and Pharmaceutical Chemistry, Technische Universität Braunschweig, D-38106 Braunschweig, Germany*

**Correspondence to:** Gerd Bendas, Department of Pharmacy, University of Bonn, An der Immenburg 4, 53121 Bonn, Germany. E-mail: gbendas@uni-bonn.de; ORCID: 0000-0002-8667-7201

**Supplementary Figure 1:** Relative expression of Trx in untreated cell pairs, normalized to W1 or A2780, respectively. Statistical analysis was performed by unpaired t-test (asterisks indicate statistical significance *p < 0.05).

|  |
| --- |

**Supplementary Figure 2:** The impact of preincubating A2780 and A2780cis cells with inhibitors for ATR (elimusertib), ATM (AZD1390), Wee1 (adavosertib), or CHK1 (SCH900776) on the cytotoxicity of auranofin (**A**) and MC3 (**B**). Data represent n = 3 samples ± SD. For statistical analysis one-way ANOVA following Tukey’s test for multiple comparisons was used.

**Supplementary Figure 3:** Relative expression of ERK 1/2 and pERK 1/2 in A2780 and A2780cis cells normalized to untreated cells. Date are corresponding to representative blots in Figure 3C and represent n = 3 samples ± SD. For statistical analysis one-way ANOVA following Tukey’s test for multiple comparisons was used (asterisks indicate *p < 0.05).
